# Memory Distortion and Its Avoidance: An Event-Related Potentials Study on False Recognition and Correct Rejection

**DOI:** 10.1371/journal.pone.0164024

**Published:** 2016-10-06

**Authors:** Sara Cadavid, Maria Soledad Beato

**Affiliations:** 1 Human Cognition Lab, Research Centre on Psychology (CIPsi), Department of Basic Psychology, School of Psychology, University of Minho, Braga, Portugal; 2 Department of Basic Psychology, Psychobiology and Methodology of the Behavioural Sciences, Faculty of Psychology, University of Salamanca, Salamanca, Spain; Vanderbilt University, UNITED STATES

## Abstract

Memory researchers have long been captivated by the nature of memory distortions and have made efforts to identify the neural correlates of true and false memories. However, the underlying mechanisms of avoiding false memories by correctly rejecting related lures remains underexplored. In this study, we employed a variant of the Deese/Roediger-McDermott paradigm to explore neural signatures of committing and avoiding false memories. ERP were obtained for True recognition, False recognition, Correct rejection of new items, and, more importantly, Correct rejection of related lures. With these ERP data, early-frontal, left-parietal, and late right-frontal old/new effects (associated with familiarity, recollection, and monitoring processes, respectively) were analysed. Results indicated that there were similar patterns for True and False recognition in all three old/new effects analysed in our study. Also, False recognition and Correct rejection of related lures activities seemed to share common underlying familiarity-based processes. The ERP similarities between False recognition and Correct rejection of related lures disappeared when recollection processes were examined because only False recognition presented a parietal old/new effect. This finding supported the view that actual false recollections underlie false memories, providing evidence consistent with previous behavioural research and with most ERP and neuroimaging studies. Later, with the onset of monitoring processes, False recognition and Correct rejection of related lures waveforms presented, again, clearly dissociated patterns. Specifically, False recognition and True recognition showed more positive going patterns than Correct rejection of related lures signal and Correct rejection of new items signature. Since False recognition and Correct rejection of related lures triggered familiarity-recognition processes, our results suggest that deciding which items are studied is based more on recollection processes, which are later supported by monitoring processes. Results are discussed in terms of Activation-Monitoring Framework and Fuzzy Trace-Theory, the most prominent explanatory theories of false memory raised with the Deese/Roediger-McDermott paradigm.

## Introduction

Memory distortions have been widely investigated during the last several decades (see [1,2] for reviews). Specifically, memory researchers have long been intrigued by the extent of similarities and differences between true and false memory at a behavioural [1,2], physiological [3], and neural level (see [4] for review). In this sense, efforts have been made to identify and describe the neural correlates of true and false recall and recognition using techniques such as *Positron Emission Tomography* (PET) [5], *Functional Magnetic Resonance Imaging* (fMRI) [6–19], *Near InfraRed Spectroscopy* (NIRS) [20], or *Event-Related Potentials* (ERP) [21]. However, little attention has been paid to the processes that underlie correct rejection of nonpresented but related lures in a recognition task, a decision that allows false memory avoidance. Correctly rejecting related lures constitutes a form of successful memory retrieval and, as such, deserves to be explored in detail [19]. The current study aimed to provide ERP evidence of the processes involved both in committing and avoiding false memories. To this end, we used an improved variant of the Deese/Roediger-McDermott (DRM) paradigm, a widely employed experimental procedure to induce false memories.

ERP studies on recognition memory have shown that ERPs produced by correctly judged “old” stimuli usually show a more positive-going deflection than the ERPs elicited by correctly judged “new” stimuli [22]. These so-called old/new effects refer to the significant differences between the activity associated with correct responses to previously studied or “old” items (i.e., true recognition) and the activity related to correct responses to nonpresented “new” items processed for the first time in the test phase (i.e., correct rejection of new items) [23,24]. It is assumed that the difference between these two types of activities constitutes an electrophysiological index of the processes that is associated with a correct memory retrieval [25].

According to dual-process models of recognition memory, recognition performance is the result of the collaboration of two different types of processes: recollection and familiarity [26–28]. The *Recollection* process is thought to be similar to that used in free recall, as it implies the retrieval of contextual details from the moment a certain event was previously encoded [27,29]. In this process, memory judgements are made based on the retrieval of qualitative information, therefore involving the conscious recollection of the prior occurrence of a certain event [22,30]. For its part, *familiarity* is considered the process that provides a quantitative basis for making recognition judgements [22]. As opposed to recollection, familiarity memory process does not bring specific information about the prior event, such as the encoding context. Instead, the familiarity process produces a nonspecific sense of a certain event which was previously experienced [4]. Furthermore, in episodic memory tasks, a third process is often reported, commonly known as *monitoring* process, which is engaged on the memorial evidence -or its lack thereof- for a particular event. Thereby, the monitoring process in this type of task is considered a post-retrieval monitoring, taking place only after the retrieval efforts are made. The monitoring process is thought to operate with both memorial evidence and task demands in order to produce the behavioural responses [31].

Analysing the old/new effects found in ERP studies with recognition memory tasks, it is possible to distinguish three main memory-related components during recognition: an early frontal old/new effect (FN400), a left-parietal old/new effect, and a late right-frontal old/new effect. Generally speaking, these components are characterized by more positive-going waveforms for true recognition than for correct rejection of new items [24,32]. The first old/new effect (FN400) is described as a negative frontally distributed component, peaking at about 400 ms after stimulus presentation (300–500 ms), and it is thought to be related to familiarity-based recognition ([24,33–35]; but see [36,37], for a discussion). This early effect has been found both in left-frontal electrodes [32] and medial-frontal sites [38]. The second old/new effect, a left-parietal component (500–800 ms) [39–42], has been related to recollection-based memory processes [43–45]. The third old/new effect described in recognition memory literature is a late right-frontal component, which has been related to strategic retrieval efforts [42,46,47] and, more recently, to post-retrieval evaluation processes involved in decision making processes [48]. This late post-retrieval effect indexes monitoring processes [21,42,44,49–57], starts around 800 ms post-stimulus and continues up to 1900 ms post-stimulus [29,32,48].

### False recognition and ERP

False memory research involving ERP has employed different types of stimuli: categorical lists [38,54,58]; categorically related pairs of words [59]; pictures or drawings [29]; or associative lists, as in the DRM paradigm [60–62].

#### DRM paradigm: Theoretical accounts

In the DRM paradigm, participants study lists of words (e.g., *low*, *clouds*, *up*, *tall*, *tower*, *jump*, *above*, etc.) associated with another, nonpresented, related lure (e.g., HIGH). In a subsequent memory test, this related lure is often falsely recalled and/or recognized (false memory) along with the presented items (true memory) [63,64].

There are two main theoretical approaches that account for memory illusions raised with the DRM paradigm: the Fuzzy-Trace Theory (FTT) [65,66] and the Activation-Monitoring Framework (AMF) [67]. Both theories converge in postulating the interplay of two processes (error-inflating and error-editing) on memory tasks. Regarding true memory, these processes would work together to boost performance. However, error-inflating and error-editing processes work in opposition to elicit false memories [68]. The FTT states that studying several words that are all linked to a related lure produces a gist representation of the list. As the related lure matches the gist representation of the list, in the memory task, participants experience a strong feeling of familiarity regarding the related lure (phantom recollection), even though it was never presented before (error-inflating processes) [65,68,69]. In other words, within the FTT, gist and familiarity seem to “share similar theoretical constructs” [70], suggesting that familiarity would contribute to gist more than recollection does ([27], but see [71,72]). Consequently, participants that endorse a related lure as if it had been studied would be unable to retrieve specific information regarding the related lure [4]. In the FTT, the ability to avoid false alarms of related lures is referred to as recollection rejection (error-editing processes). In this context, a false memory could be avoided through the retrieval of verbatim traces of the actual studied words. As these verbatim traces do not match the related lure, it would be possible to edit out the memory illusion [66].

For its part, within the AMF, the two processes are called activation (error-inflating) and monitoring (error-editing). According to the AMF, during the encoding of studied words, their related lure representation is activated due to an automatic activation of pre-existing associations that link studied words to the related lure (for evidence of activation at the memory task, see [73]). The activation of the related lure at study would allow the association of the related lure’s representation with the encoding context (error-inflating processes) [4]. At test, related lure representations are highly active, producing the experience of having been studied before. The AMF postulates that the more the activation increases, the more the false memory will be found [74]. Within the AMF, monitoring processes intervene both at encoding and at retrieval in order to avoid false memories (error-editing processes). At encoding, monitoring processes contribute to differentiate external events (i.e., presentation of the studied words) from internal events (i.e., thoughts produced by the studied word, such as the mental generation of the related lure). At retrieval, monitoring processes allow telling thoughts apart (i.e., related lure) from the experienced events (i.e., studied words) [74,75].

An important finding in DRM literature is that a full range of experimental procedures (e.g., explicit warnings, remember/know judgements, or distinctive encoding, among many others) has shown that the memorial evidence for falsely recognized related lures is extremely compelling [64,76,77]. This finding suggests the existence of false recollections when related lures are endorsed (see [4] for review). Even clearer evidence of the existence of actual false recollections of false memories is provided by studies where the retrieval of the encoding context is analysed. In these studies, participants were asked to retrieve specific information about the encoding context. Results have shown that participants not only retrieve the context for studied words (i.e., real events), but also claim to retrieve contextual details (i.e., details that are present during the encoding of studied words) for falsely recalled/recognized related lures (i.e., nonoccurred events) [4,78,79]. As opposed to what occurs with true memory [80], apparently, no item-specific details of the related lures are processed at encoding. Consequently, the existence of recollection-like memory illusions is extremely intriguing. In the absence of such specific details, what are we remembering when we produce a false memory? Moreover, participants report having strong beliefs regarding the occurrence of the related lures [4]; they actually believe they are recollecting a real event from the study list [4,64,78,81]. In other words, behavioural evidence indicates that when participants commit a false memory in the DRM paradigm, they could be experiencing a false recollection, characterized by being “accompanied by retrieval of highly specific information that was encountered during the encoding” [4].

Despite the fact that the FTT and the AMF share a dual-process perspective to explain false memory phenomena, these theories differ in their considerations regarding the involvement of recollection processes. Whereas the FTT posits that false memories are produced more by familiarity-driven processes [4], the AMF suggests that false memories can be underlain by recollection-driven processes [4]. As noted above, from the perspective of the AMF theory, related lures that are highly activated during encoding can become associated with the encoding context, increasing their chances of being falsely retrieved. This association would explain the fact that participants are able to report item-specific details about the related lures [4], an important characteristic of recollection-based memory, making the AMF account for these data.

#### ERP components in false memory: Previous findings, theoretical predictions and methodological issues

Paradoxically, although the DRM paradigm has been widely used in behavioural experiments for the last two decades (e.g. see [82], for reviews see [1,2,4]), ERP studies conducted with the DRM paradigm are not that numerous. Moreover, old/new effects found in these investigations have not been consistent across different studies. For example, regarding the FN400 effect, some DRM studies have found that the neural signature associated with the correct rejection of new items is more negative than the waveforms related to both true and false recognition [53,83]. In other words, these studies have found old/new effects in both true and false recognition, where true and false recognition waveforms are undistinguishable among them and significantly more positive than the correct rejection of new items activity. The evidence that false recognition of the related lure differs from correct rejection of new items would reflect the existence of familiarity-based recognition in false memories, just as it occurs in true memories [24]. Nonetheless, in some studies, the waveform during false recognition of the related lure was indistinguishable from correct rejections of new items, while true recognition still showed a more positive waveform [21].

Continuing with ERP evidence on false memory, on the left-parietal old/new effect, different patterns of results have been found. On the one hand, some studies have shown that the waveforms associated with both true and false recognition are more positive than the signals triggered by correct rejection of new items (e.g. [53,60,62,84]; see also “random” condition in [84]). This finding suggests that recollection processes are found in both true and false recognition. However, other studies have found that only true recognition shows a significant difference with correct rejection of new items, meaning that true but not false recognition presents this parietal old/new effect [21,83]. In other words, ERP studies show inconsistent evidence on the involvement of recollection-based processes in false memories, so its role remains unsettled. As these processes have important implications in unravelling the nature of memory distortions [4], they deserve to be thoroughly explored. Furthermore, getting to know recollection processes is also important because they possibly alter the post-retrieval monitoring processes, doing it at least in two different ways. On the one hand, monitoring processes might be less likely to be initiated if sufficient recollected details are retrieved [85]. On the other hand, if participants base their decisions on the recollection of details, post-retrieval monitoring processes may be always engaged [86].

Finally, DRM evidence of the late right-frontal old/new effect has not been conclusive either. Whereas some research has shown that both true and false recognition waveforms are more positive-going than correct rejection of new items [21,53,60], other studies have not found this pattern [83].

All these contradictory findings in ERP research are puzzling, especially regarding the left-parietal effect, as the existence of recollection processes associated with DRM false memories is highly supported by behavioural results (see [4] for review).

Regarding the ERPs of false recognition, the AMF and the FTT make different predictions. The FTT posits that false memories are originated by familiarity-driven processes, so it predicts that false recognition would show FN400 patterns similar to those found in true recognition. The FTT would not anticipate the presence of left-parietal effect for false memory. Instead, the AMF posits that false recollections underlie false memories. Therefore, within the AMF, parietal old/new effects would be expected not only for true, but also for false recognition. Since both the AMF and the FTT propose the intervention of monitoring processes, one could expect both theories to find the late right-frontal old/new effect for both true and false recognition.

The lack of consistency in ERP results obtained with the DRM paradigm might be related to the stimuli employed in those studies [4]. ERP research with the DRM paradigm has faced an important methodological limitation: the poor signal-to-noise ratio of the trials for the related lures. Signal-to-noise ratio rises as a function of the square root of the number of trials, and it is used to measure the quality of the signal [87]. Artifact-free ERP waveforms are obtained by averaging numerous EEG segments [87]. Standard DRM lists are composed of 15 studied words associated with just one related lure. Consequently, in ERP studies using the DRM paradigm, the related lure trials would not be enough to perform robust statistical analyses of neural activity related to false recognition [60,88,89]. In an attempt to solve the problem of having few trials for related lures, most DRM studies that use ERP include some kind of variation on the standard procedure to increase the number of trials for related lures per study list. A common procedure to raise the number of related lures is to consider related lures to be not only the original related lure, but also the first associate(s) of the list [21,53,62,90]. In these cases, not only the actual related lure is not presented at the study phase–just like in the standard DRM procedure–, but also the first associates of the list do not appear at the encoding phase. In the subsequent recognition memory task, participants are presented with (1) related lures, (2) associates that were studied, and (3) associates that were not studied and are considered related lures. However, this variation of the DRM paradigm disrupts the whole associative structure of the lists. In fact, in this DRM variant, the association between the “new” related lures (associates not presented at study) and the remaining associates is not controlled or even known. Therefore, it might be the case that this DRM variant could lead to less robust false recollection signals so that in some studies the false recognitions would be due to familiarity-based errors while in other studies the false recognitions would be due to recollection-based illusory errors.

As far as we know, only one ERP study has employed DRM lists with more than one actual related lure [60], therefore improving the signal-to-noise ratio in false recognition ERP data. In the present study, we sought to obtain robust ERP data with the aim of shedding light on the nature of neural signatures elicited by true and false memories. In order to do so, we maximized the signal-to-noise ratio of our materials by increasing the number of averaged trials. Specifically, we built new DRM lists composed of six associates (studied words), each of which was simultaneously linked to three (instead of just one) related lures, following the criteria proposed by a previous study [89]. Contrary to previous solutions, in these lists, all the three related lures are actually associated to the studied words, making possible to know and to control the nature of the associations between the related lures and the associates. Also in an attempt to improve the signal-to-noise ratio, this study included a large number of participants.

Another possible reason for the lack of consistency in the results could be the type of association of the lists used in the studies. Most DRM research employs word lists created by Roediger and McDermott [64], which were constructed according to forward associative strength (FAS; i.e., the related lure elicited the associates in a free association task). ERP experiments conducted with the DRM paradigm have also systematically used FAS lists [21,53,62,90]. However, DRM research has pointed out the importance of using backward associative strength (BAS) to build the lists (i.e., where the associates produce the related lure in a free association task) for studying false memories in the DRM paradigm [74,91–93]. In order to follow previous recommendations, in the present study, our lists were built taking into account the backward associative strength.

#### Resisting false memory: Correct rejection of related lures

As seen above, in false memory studies where the ERP technique is employed, researchers analyse the neural activity triggered by studied words correctly answered (i.e., true recognition), false alarms to related lures (i.e., false recognition), and correct answers to unrelated new items (i.e., correct rejection of new items). However, analysing just these three types of activities raises an essential question: What are the differences between the neural processes associated with false memories and correct rejections to related lures? That is, why are some related lures falsely remembered whereas other related lures are correctly rejected? To answer these questions, we believe it is crucial to follow the proposal of previous studies where a fourth type of neural activity is analysed: the activity triggered by correct rejection of related lures [19,29,94]. Identifying and describing the electrophysiological activity of avoiding false memories seems essential to obtain a clear picture on the processes involved in both successful and unsuccessful memory [19].

Correct rejection of related lures has been examined in a few other ERP memory studies with stimuli as pictures or photos [29,54] or common concrete nouns in singular and plural [94]. Surprisingly, however, a single DRM paradigm study has analysed ERPs of correct rejection of related lures [62]. Wiese and Daum’s [62] work focused only on one time window (400–700 ms), finding differences between false recognition and correct rejection of related lures mainly at prefrontal and frontal sites (and only in good performers). At a left-parietal region, the correct rejection of new items and the correct rejection of related lures signatures were significantly more negative than the waveform associated with false recognition. These findings provide important evidence, constituting an initial approach to study the activity of correct rejection of related lures in the DRM paradigm. However, in Wiese and Daum’s study, no information about familiarity and monitoring processes is provided, thereby ignoring these important recognition memory effects. Furthermore, in order to increase the number of trials for related lures in the recognition test, Wiese and Daum [62] used some associated words as related lures, which, as we noted above, could present an important methodological limitation. Therefore, neural signatures associated with correct rejection of related lures have not been thoroughly studied in DRM studies, and their exploration could be fundamental to understand the nature of false memories.

#### The present study: What, why, how and expectations

The present study addressed the general question of how we can distinguish successful from unsuccessful memories, with a special interest in understanding the processes entangled in avoiding false memory formation and production. Comprehending how false memories are produced (i.e., false recognition) or avoided (i.e., correct rejection of related lures) could provide a better understanding of human memory functioning. Our study aims to produce data in this direction. Specifically, we provided and compared ERP data for four types of brain activity: True recognition, False recognition, Correct rejection of new items, and, interestingly, Correct rejection of related lures. All these activities were analysed in the three old/new effects (FN400, left-parietal and late right-frontal) related to three major memory processes (familiarity, recollection, and monitoring, respectively). Despite the undeniable importance of all these processes to understand false memories, we were especially intrigued by the role of recollection-based processes in both false memory formation and its avoidance, an issue that remains unclear [4].

Previous ERP studies on false memories with the DRM paradigm do not reach an agreement on the old/new effects related to familiarity, recollection, and monitoring processes. Moreover, the neural processes related to correctly rejecting related lures remain understudied and the few studies that have tried to unravel the neural mechanisms that underlie false memories raised with associative lists could be improved. To overcome difficulties found in previous research, we built our DRM lists according to the backward associative strength and included three related lures per list. These materials preserved the associative structure of standard DRM lists and assured a better signal-to-noise ratio for the ERPs of related lures.

Regarding the familiarity-related component FN400, we expected similar patterns for True and False recognition. This finding would be in line with the FTT and, due to its focus on associative processes, the AMF could also accommodate this prediction. Most importantly, we predicted that the Correct rejection of related lures would also present a FN400 effect. Even though these particular lures are not falsely recognized, they all share some associative and semantic features with studied words, which would lead to automatic reinstatement of their relational representations. In this case, regardless of being falsely recognized or correctly rejected, related lures would present a FN400 effect. In this first time window, only Correct rejection of new items would not trigger the nonspecific sense of familiarity described above. Concerning the recollection-related component, as mentioned earlier in this paper, previous false memory research has shown mixed results with regard to whether or not false recognition shows the left-parietal old/new effect. Since the memory illusions raised with the DRM paradigm seem to imply the retrieval of encoding context–and, therefore, false recollections–, parietal old/new effects would be expected not only for True, but also for False recognition. To put it differently, based on the outcomes of behavioural studies [4,64,78,81], one might expect both True and False recognition to show left-parietal old/new effects. If this were the case, only the AMF, and not the FTT, could account for the results, as only the AMF posits that false recollections underlie false memories. In other words, the AMF and the FTT differ on their predictions about the appearance of left-parietal effects for false recognition. We wanted to explore this component in order to address whether false memories entail false recollections. Regarding Correct rejection of related lures, we did not expect to find a left parietal old/new effect, as no recollection process is associated to this type of activity. Finally, concerning the third component (associated to monitoring processes), late right-frontal effects were expected just for True and False recognition electrical activities. We anticipated that the late right-frontal effect would show indistinguishable patterns for True and False recognition, which we expected to show more positive waveforms than Correct rejection of related lures and new items. These effects would be reflecting the engagement of post-retrieval monitoring processes, responsible for assessing whether the memorial evidence obtained for the words is enough to claim their previous occurrence.

In summary, for the first time in associative illusions of memory literature, we obtained reliable data for True and False recognition, and for Correct rejection of new items and Correct rejection of related lures for the three core ERP components in memory research (FN400, left-parietal, and late right-frontal old/new effects), thus contributing to clarify the nature of human memory distortions.

## Methods

### Participants

The Bioethics Committee of the University of Salamanca acknowledged that this research fulfilled all the bioethics requirements and approved the study, which was conducted in autumn 2014. Eighty-nine University of Salamanca undergraduate students, native Spanish speakers (60 women; *M* age = 22.9 years, *SD* = 3.0), participated voluntarily, signed an informed consent form to participate in an ERP study on recognition memory and received one course credit for their participation. Seven participants were not included in the ERP analyses due to technical problems. Participants with neurological or psychological disorders or under the effects of psychotropic substances were excluded from participation in the study. Full behavioural and ERP data can be consulted in the two sheets of the S1 Table.

### Material

Thirty-two word DRM lists were built based upon free-association norms in Spanish [95]. Each list was composed of six associates that were simultaneously related to three critical lures (i.e., they were related via backward associative strength, or BAS). The BAS values per list (*BAS list*) were calculated as the sum of the BAS values for the three related lures [89] (range: 0.20–1.60, *M* = 0.85, *SD* = 0.49).

For the application of the study, the pool of lists was distributed in two groups of sixteen lists, avoiding word repetition within each group (neither associate nor related). Therefore, each participant studied sixteen lists, presented in a male voice. Studied words within each DRM list were arranged in decreasing order of BAS and the order of the lists was randomized.

Stimuli were presented using E-Prime 1.0 [96]. The EEG recording was acquired using the BrainVision Recorder software (v.1.03, Brain Products GmbH). Raw EEG data were processed offline using BrainVision Analyzer (v.1.05, Brain Products GmbH, Gilching, Germany).

The recognition memory test included all the studied words (96 items), as well as their corresponding related lures (48 items). The test also included 48 unrelated new items, extracted from other DRM lists [89].

### Procedure

In the individual sessions, after signing the consent form, participants sat in front of a computer at a distance of approximately 60 cm from the screen. Participants were informed they were participating in a memory and math skills experiment.

In the study phase, participants listened to 16 lists of words and were instructed to study each word for a subsequent memory test. Words were presented every 2000 ms. After studying each list, solved arithmetic operations were shown on the screen. Participants had to decide whether they were correct or not by pressing “Yes” or “No” keys. This task was self-paced, lasted 20s and provided feedback on accuracy and reaction time. The study phase concluded when all the 16 lists and the 16 math blocks were presented.

**Fig 1:**
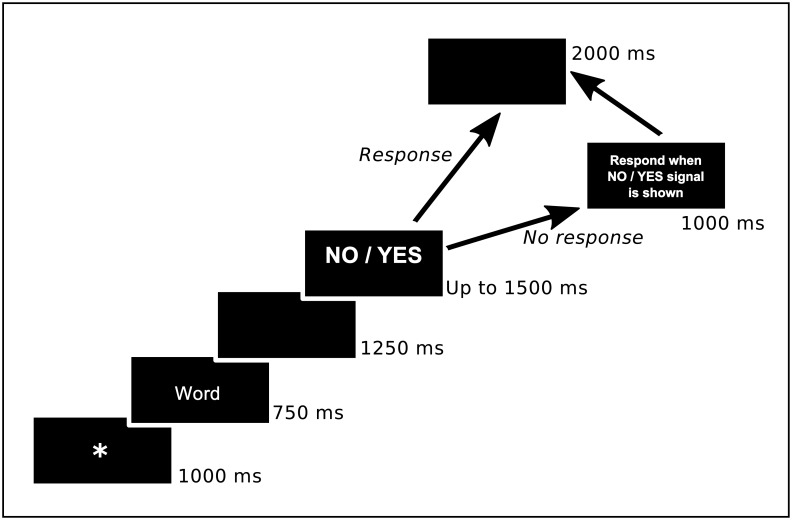
Stimuli presentation for each recognition test trial.

In the recognition memory test, participants were informed that they would be presented with words one at a time on the computer screen. Their task consisted of deciding whether each word was previously studied (by pressing the “Yes” key to indicate it was an OLD word) or not (using the “No” key to indicate it was NEW word). They should respond only when a NO/YES signal was shown in the screen (recognition test trials are described in detail in Fig 1). The items included in the recognition test were randomly presented. After the recognition test, participants were debriefed, thanked and dismissed.

### EEG recording

Throughout the experiment, the electroencephalogram (EEG) was recorded from 61 sintered Ag/AgCl electrodes embedded in an elastic cap (Electro-Cap International, Eaton, Ohio, USA) according to the 10/20 system. Reference electrodes were placed on both earlobes. To monitor vertical eye movements, two electrodes were used at the left supra- and infraorbital sites. To monitor horizontal eye movements, two electrodes were placed at the right and left external canthi sites. All the electrodes were connected to a DC amplifier (QuickAmp 136 of 128 EEG channels, Brain Products GmbH, Gilching, Germany). EEG signals were continuously recorded, sampled at a rate of 500 Hz. Interelectrode impedance was kept below 8 kΩ. Participants were asked to minimize their movements during the recording session.

### EEG data analyses

In the recognition test, EEG data were segmented from 300 ms prior to stimulus onset, to 4000 ms after stimulus onset, filtered (bandpass filter of 0.1–35 Hz, 12 dB/oct) and baseline corrected using the first 300 ms. Ocular artifacts were corrected using the method of Gratton, Coles, and Donchin [97]. Segments containing activity greater than ±80 μV were rejected. Artifact-free segments were averaged across the electrodes of interest, across the a priori time bins of interest. There were four types of activities of interest: True recognition, False recognition, Correct rejection of related lures, and Correct rejection of new items. In order to study the FN400, the left-parietal, and the late right-frontal old/new effects, different electrodes (and epochs) were taken into account in the ERP analysis: F1-F3 (300–500 ms), CP3 (500–800 ms), and F6-F8 (1000–1500 ms), respectively.

## Results

For the within-subjects ANOVAs, degrees of freedom were corrected for sphericity violations using the Greenhouse-Geisser estimator. Effect sizes are reported with partial eta-squared values (*η*^*2*^_*p*_).

### Behavioural analysis

A one-way repeated measures ANOVA (Type of item: studied words, related lures, new items) was conducted to explore whether there was a false recognition effect (false alarms to related lures significantly higher than false alarms to new items). Results showed a significant effect of Type of item, *F*(1.865, 164.162) = 444.953, *p* < .001, *η*^*2*^_*p*_ = .835 (see Table 1). A Bonferroni post-hoc analysis revealed that correct responses to previously studied words (i.e., true recognition) were higher than false alarms to related lures (i.e., false recognition), with a statistically significant difference of 25.99 (95% CI, 21.04 to 30.94, *p* < .001). True recognition was also higher than false alarms to new items, showing a difference of 54.16 (95% CI, 49.79 to 58.53, *p* < .001). More importantly, false recognition was significantly higher than false alarms to new items, with a difference of 28.17 (95% CI, 24.25 to 32.09, *p* < .001), confirming that related lures produced above-baseline levels of false recognition (see Table 1).

**Table 1:** Behavioural data of “old” responses to each Type of word (percentages).

Type of word	Mean	SD	Minimum	Maximum
Studied word (True recognition)	65.30	14.33	26.67	95.56
Related lure (False recognition)	39.31	14.65	6.67	67.78
New item (Unrelated false alarms)	11.14	9.54	0.00	43.75

### EEG analysis

**Fig 2:**
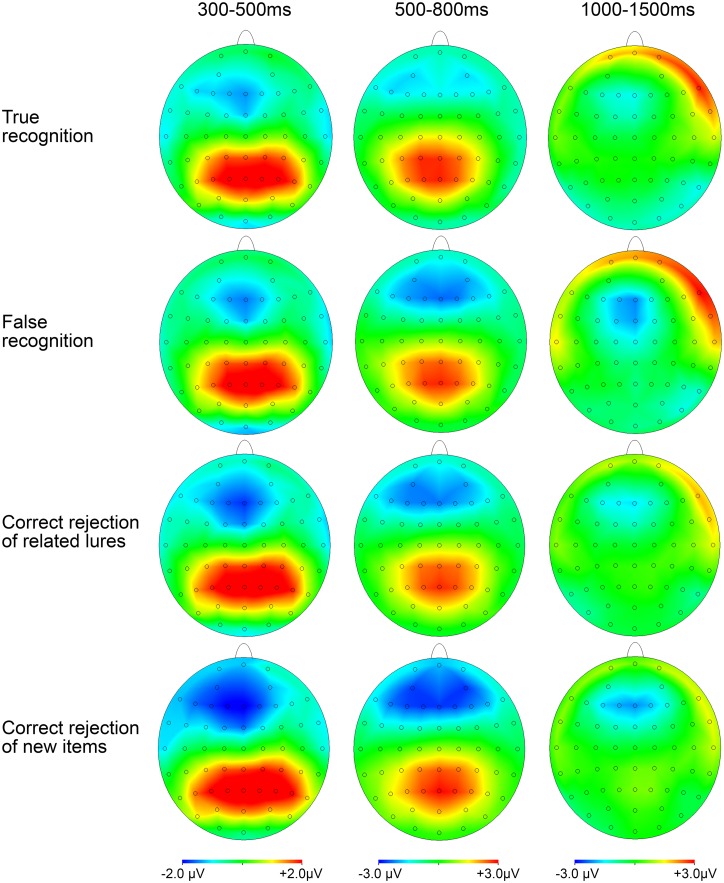
Scalp topography distributions in the three time windows analysed, considering each Type of item.

Scalp topography for each time window and type of item analysed can be found in Fig 2.

#### FN400 old/new effect (300–500 ms)

**Fig 3:**
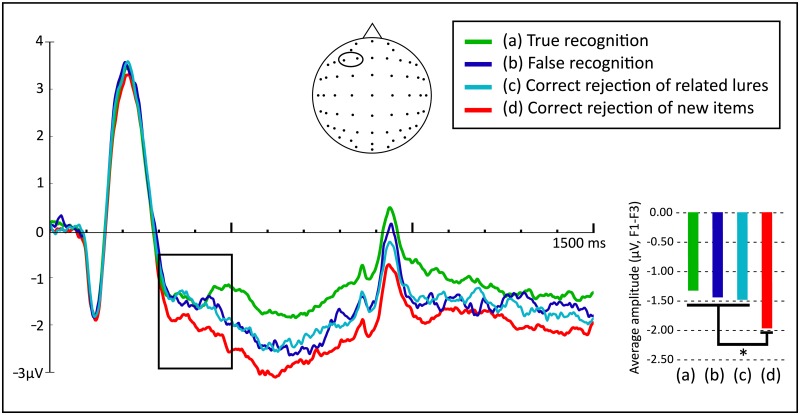
Cortical responses in microvolts (μV, electrodes F1 and F3) to True recognition, False recognition, Correct rejection of related lures, and Correct rejection of new items between 0 and 1500 ms during the recognition test. The highlighted area corresponds to the interval where the FN400 old/new effect was explored (300–500 ms, *p < .05).

With the aim of exploring whether there were significant differences between electrical activities shown by the Type of activity, a one-way repeated measures ANOVA was conducted (Type of activity: True recognition, False recognition, Correct rejection of related lures, Correct rejection of new items). Results showed a significant effect, *F*(3, 243) = 6.409, *p* < .001, *η*^*2*^_*p*_ = .073. A Bonferroni post-hoc analysis revealed no significant differences between True recognition (*M* = -1.34, *SD* = 2.20), False recognition (*M* = -1.46, *SD* = 2.30) and Correct rejection of related lures (*M* = -1.49, *SD* = 2.27) waveforms (*p* = 1 for the three comparisons). However, True recognition was more positive than Correct rejection of new items activity (*M* = -1.95, *SD* = 2.34) (Fig 3), with a statistically significant difference of 0.612 (95% CI, 0.252 to 0.973, *p* < .001). False recognition and Correct rejection of related lures activities also differed from Correct rejection of new items, presenting differences of 0.487 (95% CI, 0.017 to 0.958, *p* = .038) and 0.465 (95% CI, 0.069 to 0.862, *p* = .013), respectively (Fig 3). In other words, there was FN400 old/new effect for True recognition and, more notably, this early FN400 effect, associated with familiarity, was found in both False recognition and Correct rejection of related lures. That is, FN400 old/new effect occurred in related lures regardless of whether they were falsely recognized or correctly rejected (Fig 3).

#### Left-parietal old/new effect (500–800 ms)

**Fig 4:**
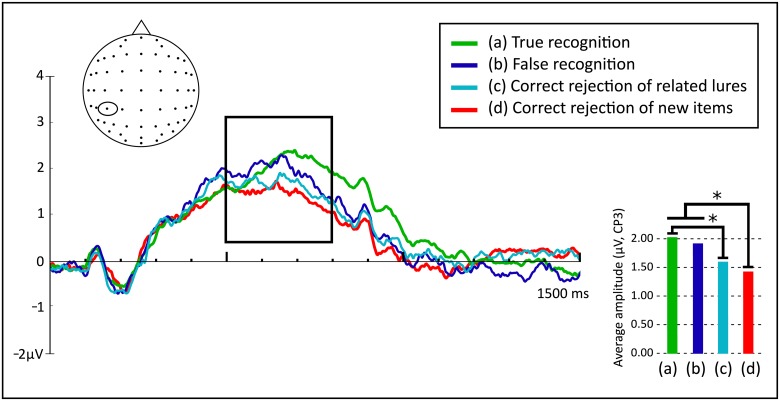
Cortical responses in microvolts (μV, electrode CP3) to True recognition, False recognition, Correct rejection of related lures, and Correct rejection of new items between 0 and 1500 ms during the recognition test. The highlighted area corresponds to the interval where the left-parietal old/new effect was explored (500–800 ms, *p < .05).

A repeated measures ANOVA (Type of activity: True recognition, False recognition, Correct rejection of related lures, Correct rejection of new items) showed significant differences between levels, *F*(2.657, 215.202) = 8.412, *p* < .001, *η*^*2*^_*p*_ = .094. A Bonferroni post-hoc analysis showed that True recognition (*M* = 2.02, *SD* = 1.79) and False recognition (*M* = 1.89, *SD* = 2.00) patterns did not differ significantly (*p* = 1). Furthermore, activity associated with True recognition was significantly more positive than activity associated with Correct rejection of new items (*M* = 1.44, *SD* = 1.73) (Fig 4), with a difference of 0.581 (95% CI, 0.287 to 0.875, *p* < .001). False recognition was also more positive than Correct rejection of new items, with a difference of 0.456 (95% CI, 0.058 to 0.853, *p* = .016). False recognition and Correct rejection of related lures (*M* = 1.61, *SD* = 1.95) activities did not differ significantly (*p* = .273). As opposed to what was found in the FN400, at the left-parietal component, Correct rejection of related lures and Correct rejection of new items activities did not differ among them (*p* = 1). Therefore, left-parietal old/new effect was present both for True recognition and False recognition, but not for related lures that were correctly rejected. Finally, True recognition differed significantly from Correct rejection of related lures (Fig 4), with a difference of 0.408 (95% CI, 0.107 to 0.709, *p* = .003).

#### Late right-frontal old/new effect (1000–1500 ms)

**Fig 5:**
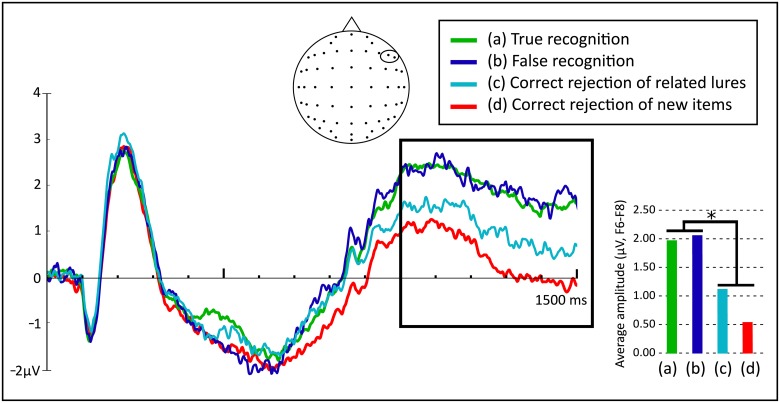
Cortical responses in microvolts (μV, electrodes F6 and F8) to True recognition, False recognition, Correct rejection of related lures, and Correct rejection of new items between 0 and 1500 ms during the recognition test. The highlighted area corresponds to the interval where the late right-frontal old/new effect was explored (1000–1500 ms, *p < .05).

Again, a repeated measures ANOVA (Type of activity: True recognition, False recognition, Correct rejection of related lures, Correct rejection of new items) indicated a significant effect, *F*(2.656, 215.143) = 15.102, *p* < .001, *η*^*2*^_*p*_ = .157. Bonferroni post-hoc tests showed no differences between True recognition (*M* = 1.98, *SD* = 2.52) and False recognition (*M* = 2.04, *SD* = 2.95) waveforms (*p* = 1). Also, there was more positivity of True recognition and False recognition than Correct rejection of new items (*M* = 0.53, *SD* = 2.20) (Fig 5), with differences of 1.459 (95% CI, 0.860 to 2.058, *p* < .001) and 1.516 (95% CI, 0.700 to 2.333, *p* < .001), respectively. For its part, Correct rejection of related lures (*M* = 1.11, *SD* = 2.70) activity did not differ significantly from activity associated with Correct rejection of new items (*p* = .175). True recognition and False recognition activities turned out to be more positive than the Correct rejection of related lures mean amplitude, with differences of 0.869 (95% CI, 0.115 to 1.623, *p* = .015) and 0.926 (95% CI, 0.154 to 1.699, *p* = .010), respectively. In summary, late right-frontal old/new effects were found for both True and False recognition but, again, not for the related lures that were correctly rejected (Fig 5).

## Discussion

The present study addressed the general question of how we can distinguish successful from unsuccessful memories using ERPs; a matter that remains a major topic of debate. Indeed, processes involved in avoiding false memory formation and production had not been closely examined. Previous studies on true and false memories showed no consistency and presented some methodological issues that we aimed to improve. That is why, in our study, following the approach employed by Beato et al. [60], we built DRM lists with three–and not just one–actual related lures, constructed our lists taking into account the BAS–and not the FAS–, and included a considerable number of participants.

With the main goal of examining underlying processes of successful and unsuccessful retrieval, we explored the three main recognition memory-related ERP components: the FN400, the left-parietal, and the late right-frontal old/new effects, associated with processes of familiarity, recollection, and monitoring, respectively [24,58,98,99]. More importantly to our goal, we analysed not only the electrical activity associated to True recognition, False recognition, and Correct rejection of new items, as in previous studies, but also, for the first time in the DRM literature, we systematically explored waveforms of Correct rejection of related lures.

At a behavioural level, our findings indicate the existence of a robust false recognition effect, as false alarms to related lures were significantly higher than false alarms to new items. This result replicated other previous DRM studies with one [64] (for reviews see [1,2]) and three related lures [60,89,91] per list, assuring the adequacy of the lists to induce memory distortions.

Regarding ERP data, first we found early frontal old/new effects (FN400) for True recognition, False recognition and Correct rejection of related lures. Second, left-parietal old/new effects were found for both True and False recognition activities, whereas Correct rejection of related lures did not show this old/new effect. Third, our data pointed out the existence of late right-frontal old/new effects for True and False recognition. Again, Correct rejection of related lures did not show this old/new effect.

### Previous studies: overcoming methodological limitations

Previous ERP false memory research conducted with the DRM paradigm has shown inconsistencies across studies on whether false memory signals are similar to true memory signatures. Some studies have found evidence that false memory elicits signatures that are indistinguishable from true memory at certain time intervals traditionally related to recognition memory old/new effects (for similitudes in FN400 effect, see [83]; in left-parietal effect, see [60,62]; in late right-frontal effects, see [21,60]). However, other studies have found clearly dissociated patterns of neural activity for True and False recognition in those time intervals (for differentiated waveforms in FN400 see [21]; in left-parietal effect, see [21,83]; in late right-frontal effect, see [83]).

This lack of consensus in the literature about the neural activity involved in old/new effects raised with false memories could derive from methodological problems related to the materials employed in previous ERP studies [4]. To overcome these problems, our ERP study was conducted with carefully built materials in order to create an optimal tool to gather evidence on the cognitive processes implicated in true and false memories. Specifically, word lists included six words simultaneously associated with three (and not just one) related lures. Furthermore, lists were constructed using Backward Associative Strength (BAS). These lists served to carry out a robust statistical analysis of the brain activity associated with True and False recognition using the DRM paradigm, as they improve the signal-to-noise ratio of related lure trials. Using three-related lure lists avoided the need to increase the number of trials for related lures with strategies that are not entirely desirable, such as using the highest-ranking associates as related lures [21,53,62,90]. With these materials, we could contribute to a better understanding of the extent to which true memories are distinguished or not from false memories, as well as gain insight on how we avoid committing false memory errors. That is why we analysed not only the activity usually explored in prior ERP research on false memory (i.e., True recognition, False recognition, and Correct rejection of new items), but also the neural signature of Correct rejection of related lures. The activity elicited by correctly rejecting related lures had received little attention in ERP studies of false memories [19,29], especially in studies where the DRM paradigm is employed.

Therefore, the present study constitutes not only the first attempt to analyse the FN400, left-parietal, and late right-frontal old/new effects including the brain activity triggered by both correct and incorrect responses to related lures (Correct rejection of related lures and False recognition, respectively), but it is also the first study where brain activities are collected with material specially designed to enhance the robustness of the signals of related lures. Consequently, the results obtained in our study could be considered highly reliable.

### ERP results: old/new effects

#### FN400 effect

For the first time in ERP research on false memory with the DRM paradigm, we obtained neural activities elicited at test between 300–500 ms for both correct and incorrect answers to related lures (i.e., Correct rejection of related lures and False recognition, respectively) and compared them with the pattern of Correct rejection of new items. In addition, as in previous memory ERP research, we explored the waveforms triggered by True recognition and Correct rejection of new items. In our study, results indicated that Correct rejection of new items activity was significantly more negative than False recognition and Correct rejection of related lures, suggesting the existence of an early familiarity-based recognition process in all the related lures, regardless of whether they were falsely recognized later or not. Furthermore, True recognition also presented familiarity-based processes, as the Correct rejection of new items signature showed a more negative-going signature than True recognition waveforms [53,83].

Regarding the theoretical approach proposals, the similarity between True and False recognition at early stages of memory processes is a finding that both the Fuzzy-Trace Theory (FTT) and the Activation-Monitoring Framework (AMF) could accommodate. Instead, interpreting the similarity between False recognition and Correct rejection of related lures seems more challenging. This similarity might suggest that participants experience a sense of familiarity when presented with studied words and related lures (both subsequently falsely recognized and correctly rejected). As mentioned in the Introduction, the FTT posits that false memories are raised by a strong feeling of familiarity. Consequently, explaining why related lures that are correctly rejected in the memory test trigger familiarity-based processes is not straightforward with the FTT approach. In contrast, the AMF would claim that all the related lures, regardless of whether they are falsely recognized or not, are activated, thereby producing familiarity processes. Therefore, compared to the FTT, the Activation-Monitoring Framework seems to better account for the results in this component.

#### Left-parietal old/new effect

With respect to the left-parietal old/new effect, typically related to recollection processes, both True and False recognition activities were more positive than Correct rejection of new items in the present study. This outcome replicates some previous ERP studies [53,60,62] (see [21] for a discussion) where recollection-based recognition is found in both true and false memories. Also, this result supports the view that authentic recollections underlie false memory, providing consistent evidence with behavioural research.

Moreover, finding recollection-based recognition for False recognition endorses the AMF, which predicted that a false recollection of a related lure can occur if it is activated by processing the studied words [67]. In other words, the AMF proposed that a false memory raised with the DRM paradigm is more than a product of a strong familiarity sense, characterized by the experiencing of actual recollections. However, the FTT could not fully accommodate this result, as it suggests that false memories are more a familiarity process by-product.

By contrast with the FN400, at the left-parietal old/new effect, no differences were observed between Correct rejection of related lures and Correct rejection of new items signals, indicating that correctly rejected related lures lacked recollection, a comparison never reported before with the DRM paradigm (for a comparison with pictures see [29]). Furthermore, in our study, the Correct rejection of related lures signature presents a significantly more negative pattern than the True recognition signal at parietal sites. This finding indicates a clear differentiation between the processes associated with correctly recognizing a studied word and with correctly avoiding a false memory. In other words, successful memory seems to rely on differential neural circuits depending on the demands of the task.

Summarizing, in this component, the signals of related lures differ considerably according to their subsequent response (i.e., False recognition or Correct rejection). Specifically, it seems like correctly rejected related lures do not trigger recollection-based recognition processes, whereas falsely recognized related lures do. In other words, we compared the signals produced by both correctly rejected and falsely recognized related lures, observing that only the latter triggered recollection-based processes, as only False recognition showed significant differences with regard to Correct rejection of new items. Thus, false memories in the DRM paradigm appear to rely on recollections of events that never happened. In fact, while in the FN400 effect (indexing familiarity) False recognition and Correct rejection of related lures showed a similar pattern, in the left-parietal effect a clear difference appeared between false memory formation and avoidance. This finding suggests that there are actual memory traces underlying False recognition of related lures, which could help explain why True and False recognition become subjectively (for review see [100]) and electrophysiologically indistinguishable to participants as those in DRM studies.

Behavioural studies conducted with the DRM paradigm have repeatedly found that false recognition of a related lure entails the retrieval of specific details of the encoding of its associates [4]. However, until this study, ERP research had not provided robust support to the idea that falsely recognized related lures in the DRM paradigm elicit recollective signatures [4]. Since this study overcame methodological obstacles of previous research, the present study contributes to solve this discrepancy between behavioural and electrophysiological research. Our findings provide evidence that further support the existence of a strong relationship between the occurrence of related-lure recollection processes and committing a false memory error. Thereby, we provide ERP evidence in favour of the existence of authentic false recollections in the DRM paradigm, which is posited by the AMF.

#### Late right-frontal old/new effect

Finally, the late right-frontal old/new effect (related to monitoring processes) was analysed between 1000–1500 ms. As in previous studies [21], in the present work we found old/new effects for both True and False recognition. That is, both True and False recognition waveforms were similar and more positive-going than the Correct rejection of new items signature. As both the FTT and the AMF posit the intervention of monitoring processes to edit-out false memories, one could expect both theories to find late right-frontal old/new effects for both true and false recognition.

In contrast, the Correct rejection of related lures signal showed no differences regarding Correct rejection of new items, a comparison never reported before. It is also worth noting that the False recognition signature significantly differed from the Correct rejection of related lures pattern, showing a more positive deflection. This difference might be related to the effects found at a parietal level, where only falsely recognized, but not correctly rejected related lures, triggered recollection-based processes (just as True recognition did). It seems that false recollections could be so compelling that they generate memory traces that are indistinguishable from true recollections. Hence, when monitoring processes are engaged, true and false recollections show the same functional pattern.

#### False recognition and correct rejection signatures

Along the three analysed old/new effects, False recognition and Correct rejection of related lures showed patterns of neural activity that could help us to understand how we avoid false memories. Analysis of the early FN400 effect indicated that False recognition and Correct rejection of related lures waveforms presented a similar pattern, which was clearly differentiated from the Correct rejection of new items signal. In other words, at early stages of the recognition test, regardless of its later response (i.e., False recognition or Correct rejection), related lures triggered neural activities related to familiarity processes, just as studied words do. Nonetheless, only falsely recognized (and not correctly rejected) related lures showed a left-parietal old/new effect, indexing recollection. This outcome suggests that recollection processes contribute to the differentiation of the neural activity elicited by related lures that would be later rejected from those that would be falsely recognized. With this result, we provided evidence in favour of the view that false memories are derived from authentic false recollections.

The analysis of the late right-frontal old/new effect, related to monitoring processes, indicated that related lures would be rejected when presented with a more negative signature than the false recognition signal. Moreover, the False recognition signature presented a very much studied-like pattern, whereas the Correct rejection of related lures elicited a waveform similar to the Correct rejection of new items. These findings suggest that memory distortions can generate compelling false memory illusions. In fact, when related lures elicit recollection processes (and hence, strong memory signals), monitoring processing is similar to the true memory post-retrieval pattern. In contrast, when related lures do not present recollection processes, they are correctly rejected and monitoring-related activity has a functional pattern similar to the waveform shown by Correct rejection of new items.

In view of these results, we suggest first that associative lists do in fact activate early memory traces for all the related lures. Second, we propose that committing false memory errors seems to be related to the occurrence of false recollections, since in the absence of recollection it was possible to avoid false memory errors. Finally, we believe that when monitoring processes are engaged, true and false recollections have already generated such strong memory signals that both are processed as if they had been authentic. In contrast, related lures that do not trigger false recollections do not seem to produce strong memory signals, presenting a functional pattern as if they were new information. Taken together, our findings suggest that familiarity processes are necessary, but not sufficient to consider a particular word as studied in a recognition test. In fact, the decision of whether or not to endorse an item as studied relies more on recollection than on familiarity processes. This decision process seems to be supported by post-retrieval monitoring processes.

## Supporting Information

S1 Table Behavioural and ERP data. The behavioural data includes “old” responses to each type of word (studied word, related lure, new item). The ERP data includes electrical brain activity (in microvolts) to True Recognition, False Recognition, Correct Rejection of Related Lures, and Correct Rejection of New Items in each time window (300–500 ms, 500–800 ms, 1000–1500 ms), at the electrodes of interest.
(XLS)
